# Bright light improves sleep in patients with Parkinson’s disease: possible role of circadian restoration

**DOI:** 10.1038/s41598-020-64645-6

**Published:** 2020-05-14

**Authors:** Takuyuki Endo, Ritsuko Matsumura, Isao T. Tokuda, Tomoko Yoshikawa, Yasufumi Shigeyoshi, Koichi Node, Saburo Sakoda, Makoto Akashi

**Affiliations:** 1 Department of Neurology, Osaka Toneyama Medical Center, 5-1-1 Toneyama, Toyonaka, Osaka, 560-8552 Japan; 2 The Research Institute for Time Studies, Yamaguchi University, 1677-1 Yoshida, Yamaguchi, Yamaguchi, 753-8511 Japan; 3 Department of Mechanical Engineering, Ritsumeikan University, 1-1-1 Nojihigashi, Kusatsu, Shiga, 525-8577 Japan; 4 Department of Anatomy and Neurobiology, Kindai University, 377-2 Ohno-Higashi, Osaka-Sayama, Osaka, 589-8511 Japan; 5 Department of Cardiovascular Medicine, Saga University, 5-1-1 Nabeshima, Saga, Saga, 849-8501 Japan; 6 Organic Clinic, 3-1-57 honmachi, Toyonaka, Osaka, 560-0021 Japan; 7 Present Address: Organization for International Education and Exchange, University of Toyama, 3190 Gofuku, Toyama, Toyama, 930-8555 Japan

**Keywords:** Circadian rhythms and sleep, Diseases of the nervous system, Neurology, Diseases

## Abstract

Parkinson’s disease (PD) is one of the most common neurodegenerative disorders. Among the most common manifestations of PD are sleep problems, which are coupled with the adverse effects of dopaminergic therapies (DT). A non-pharmacological solution for these sleep problems has been sought to avoid additional pharmacological intervention. Here, we show that bright light therapy (BLT) is effective for improving sleep in Japanese PD patients receiving DT. Furthermore, experimental evaluation of peripheral clock gene expression rhythms revealed that most PD patients receiving DT who experienced improved sleep following BLT showed a circadian phase shift, indicating the existence of a correlation between circadian modulation and sleep improvement. Conversely, this result indicates that sleep problems in PD patients receiving DT may arise at least in part as a result of circadian dysfunction. Indeed, we found that chronic dopaminergic stimulation induced a rapid attenuation of autonomous oscillations of clock gene expression in *ex vivo* cultured mouse suprachiasmatic nucleus (SCN) at the single neuron level. In conclusion, BLT is a promising medical treatment for improving sleep in PD patients receiving DT. This BLT-induced improvement may be due to the restoration of circadian function.

## Introduction

Parkinson’s disease is one of the most common neurodegenerative disorders, affecting approximately 10 million people worldwide^1^. First-line treatment strategies involve implementation of dopaminergic therapies such as dopamine replacement therapy using levodopa which is converted to dopamine by enzymes in the brain^2,3^. Among the most common manifestations of PD are sleep problems, which reduce quality of life and impair daytime functioning. For example, in a large survey of non-motor symptoms, 64% of PD patients reported sleep problems, representing the second most common non-motor complaint^4^. Previous studies have suggested that PD symptoms and the adverse effects of medications may be some of the causes of sleep problems in PD patients^5–7^. Because of the risk of interference with treatment regimens, few treatment options for sleep problems in PD are available; in particular, chronic sleep problems are coupled with the adverse effects of the medications prescribed for PD. Therefore, preventing sleep problems in patients with PD requires the development of non-pharmacological approaches.

Several lines of evidence suggest that one cause of sleep problems in patients with PD is circadian dysfunction. First, dopaminergic therapy (DT)-receiving PD patients show a circadian phase advance and decrease in nighttime levels of melatonin^8,9^. Melatonin is a pineal hormone whose circadian secretion is regulated by the hypothalamic SCN, the center of the circadian clock, via a polysynaptic efferent pathway^10^. Concurrently, melatonin is thought to play a role in enhancing the robustness of suprachiasmatic circadian function by activating its receptors expressed in the SCN^10,11^. This reciprocal association between the pineal gland and SCN suggests that melatonin is an indirect but useful indicator of suprachiasmatic function. Therefore, the abnormal circadian secretion of melatonin in DT-receiving PD patients suggests that DT causes functional defects in the suprachiasmatic circadian clock. Second, recent studies have indicated that neurodegeneration affects the retina of PD patients and frequently leads to impairment of retinal ganglion cells (RGCs)^12,13^. Within the RGC population, there is a subgroup of cells that is intrinsically photosensitive and expresses the photopigment melanopsin, which is involved in circadian entrainment to light–dark cycles. Thus, the correlation between the pathology affecting RGCs and the circadian dysfunction in PD patients points to the possibility that these patients may benefit from light therapy, the efficacy of which has been well documented in the treatment of sleep and circadian disturbances in Alzheimer’s disease^14^. Indeed, a clinical study indicates that bright light therapy (BLT) is effective against sleep problems in DT-receiving PD patients^15^. Supporting this, a recent randomized clinical trial conducted a detailed evaluation of BLT-induced sleep improvement and found that BLT was significantly associated with improvements in excessive daytime sleepiness, sleep fragmentation and sleep quality^16^. The efficacy of BLT against sleep problems in DT-receiving PD patients was confirmed in an open-label, retrospective and longitudinal study^17^. In addition, another controlled exploratory trial shows that strategic application of polychromatic light to DT-receiving PD patients improved motor and non-motor symptoms including sleep problems^18^. Together, these human studies conducted to date indicate that sleep problems in PD patients may be attributable to circadian dysfunction and that BLT may be a promising non-pharmacological approach for improving sleep in these patients. In further support for these human studies, animal studies have demonstrated that dopamine receptors expressed in the SCN play a role in a photic input pathway to modulate suprachiasmatic circadian function^19,20^. This infers that chronic exposure of the SCN to excess dopamine and its derivatives causes some functional impairment of the SCN, in turn leading to the speculation that DT-induced functional impairment of the suprachiasmatic clock causes sleep problems in DT-receiving PD patients. However, the causal link between circadian function and BLT-mediated sleep improvement in DT-receiving PD patients remains weak due to the lack of any experimental data based on biological markers for the circadian clock. In addition, there is no experimental evidence on whether chronic exposure of the SCN to excess dopamine causes suprachiasmatic circadian dysfunction.

Although melatonin is a useful biological marker for the circadian clock in research settings^21^, reliable measurement requires that patients remain under dim light conditions during sample collection^22^. Importantly, because melatonin secretion is affected by aging and neurodegenerative disorders^23,24^, there is increasing concern about the limitations of inter-individual comparisons of circadian characteristics among elderly subjects with neurodegenerative disorders. Instead, clock gene transcripts can be used as alternative markers. Circadian rhythms are driven by negative feedback loops of transcription in clock gene expression^25,26^. Cell-autonomous transcriptional feedback loops are ubiquitous throughout the body, and the SCN functions as the central clock to orchestrate clock gene expression in peripheral tissues^27^. Peripheral clock gene transcripts in humans can be measured by collecting peripheral tissues such as white blood cells, oral mucosa and hair follicles^28–30^ and are useful optional markers in experimental evaluation of the human circadian clock. Previous studies using expression data of three clock genes demonstrated that sampling at 6‐hr intervals (four times a day) allowed the reliable mathematical estimation of circadian phase^28,31^. This low sampling frequency may be advantageous compared with melatonin measurement.

In the present study, we aimed to provide further evidence on whether BLT-mediated sleep improvement in DT-receiving PD patients involves functional modulation of the central circadian clock, and whether DT-induced suprachiasmatic dysfunction is a cause of sleep problems in these patients. To do this, given the circadian phase advance of melatonin secretion and increased frequency of early-morning awakening in DT-receiving PD patients, we investigated the effect of evening BLT on sleep problems. Simultaneously, we confirmed the effect of evening BLT on circadian phase by detecting peripheral clock gene expression rhythms using plucked hairs, and compared estimated circadian phases with sleep improvement. Furthermore, to obtain evidence on the negative effects of chronic dopamine exposure on the suprachiasmatic function, we performed *ex vivo* culture of mouse SCN and real-time monitoring of clock gene expression rhythms under chronic treatment with dopamine.

## Materials and methods

### Patients

Japanese PD patients with a Unified Parkinson’s Disease Rating Scale (UPDRS) part III score of 9–45 who were aged 52–80 years and receiving DT were recruited in Osaka Toneyama Medical Center. No patient had severe cataracts, which could affect retinal light input. All subjects underwent a neuro-ophthalmic examination to confirm the absence of any abnormalities. In addition, all subjects underwent the Mini-Mental State Examination (MMSE) and Alzheimer’s Disease Assessment Scale (ADAS) to confirm that cognitive function was within the normal range. These patients spent most of the day indoors. Their daily schedule was controlled during hospitalization and re-hospitalization (wake-up time, 6:00; breakfast, 8:00; lunch, 12:00; dinner, 17:30; bedtime, 22:00). Sleep was evaluated using the Epworth Sleepiness Scale (ESS) and Parkinson Disease Sleep Scale 2 (PDSS-2). This study was conducted in accordance with the Declaration of Helsinki and was approved by the institutional review boards of Osaka Toneyama Medical Center and Senri Chuo Hospital, with all participants providing written informed consent.

### Experimental design

Patients were exposed to one hour of bright light once a day during the evening hours (19:00–21:00) using a table-top illuminator emitting 5,000-lux bright light at a distance of 50 cm with a color temperature of about 6,700 K. This bright light exposure was performed every day for a period of approximately three months in total: one week during hospitalization, more than 10 weeks at home after discharge and one week during re-hospitalization. To minimize the effects of environmental factors such as ambient light, all subjects stayed in a hospital room with blackout curtains during hospitalization and followed a regular daily schedule, as described above. Patient #4 received bright light exposure for a period of eight months due to difficulties with scheduling re-hospitalization. Before and after the period of bright light exposure, patients completed the UPDRS part III, ESS and PDSS-2, and hair follicles were collected from them at approximately 6‐hr intervals around the clock.

### non-PD subjects

Japanese non-PD subjects with an ESS score of 1–9 who were aged 59–82 years and had stayed for fracture rehabilitation in Senri Chuo Hospital were recruited. These subjects spent most of the day indoors before and during hair collection. Their daily schedule was controlled during hospitalization (wake-up time, 6:00; breakfast, 8:00; lunch, 12:00; dinner, 18:00; bedtime, 22:00).

### Statistical analysis

A Wilcoxon rank sum test was used for pairwise comparisons because it was not possible to assume that the data obtained were homoscedastic and normally distributed (Table 2). P values of less than 0.05 were considered to indicate statistical significance. All described P values are two-sided. Statistical analyses were performed using JMP 11 for Windows (SAS Institute Inc., U.S.A.).

### Peripheral clock gene expression

Hair follicles were collected by gripping and tugging the hair shaft with a pair of tweezers and were quickly soaked in Lysis Solution (RNAqueous-Micro Kit; Thermo Fisher Scientific, U.S.A.). Hair follicle cells attached to the hair shafts were stored at −20 °C until RNA purification. Approximately 2–10 scalp hair follicles were required to detect clock gene expression at each sampling timepoint. To minimize skin damage, samples were obtained from different regions of the scalp. The RNAqueous-Micro Kit was used with frozen cytolysis solution to purify total RNA. After checking the quality and concentration using a NanoDrop (LMS, Japan), total RNA was reverse-transcribed using a SuperScript VILO cDNA Synthesis Kit (Life Technologies, U.S.A.), and real-time PCR was performed using a TaqMan MGB probe (Applied Biosystems, U.S.A.) and a 1/20 volume of the reverse transcription product. Data were obtained using a PRISM7300 (Applied Biosystems, U.S.A.) and corrected by *18S ribosomal RNA* (*18S-rRNA*), the expression of which is constant regardless of cell type and sampling time^32^. The sequences of the primers and probe for the *Per3*, *Nr1d1* (*Rev-erbα*), *Nr1d2* (*Rev-erbβ*) and *18S-rRNA* transcripts are listed in our previous report^28^.

### Circadian phase estimation

Time-courses of gene expression data were normalized to fit 24-h-period cosine curves with unity amplitude. The normalized gene expressions at time t were denoted as *x*(*t*), *y*(*t*), and *z*(*t*) for *Per3*, *Nr1d1*, and *Nr1d2*, respectively. Expression data for these three genes were then fitted to the following 24-h-period cosine curves under the constraint that phase difference between *Per3* and *Nr1d1* and between *Per3* and *Nr1d2* were Δ*θ*_*y*_ and Δ*θ*_*z*_, respectively:

1 $$x(t)={A}_{x}\,\cos (\omega t+\theta )+{C}_{x},$$

2 $$y(t)={A}_{y}\,\cos (\omega t+\theta +\Delta {\theta }_{y})+{C}_{y},$$

3 $$z(t)={A}_{z}\,\cos (\omega t+\theta +\Delta {\theta }_{z})+{C}_{z}.$$

For four-point experimental measurements, the cost function was defined as follows:

4 $$\begin{array}{ccc}E(\theta ,A,C) & = & {{\Sigma }_{i=1}}^{4}[{\{x({t}_{i})-{A}_{x}\cos (\omega {t}_{i}+\theta )-{C}_{x}\}}^{2}+{\{y({t}_{i})-{A}_{y}\cos (\omega {t}_{i}+\theta +\Delta {\theta }_{y})-{C}_{y}\}}^{2}\\  &  & +{\{z({t}_{i})-{A}_{z}\cos (\omega {t}_{i}+\theta +\Delta {\theta }_{z})-{C}_{z}\}}^{2}].\end{array}$$

To minimize the cost function, model parameters, {*θ*, *A*_*x*_, *A*_*y*_, *A*_*z*_, *C*_*x*_, *C*_*y*_, *C*_*z*_}, were determined using the conjugate gradient method. Empirically, the differences in peak expression time between the clock genes are known to be around Δτ_*y*_ = 4 h and Δτ_*z*_ = 2 h, which translate to phase differences of Δ*θ*_*y*_ = 2πΔτ_*y*_/24 and Δ*θ*_*z*_ = 2πΔτ_*z*_/24, respectively. By varying the phase differences in the range of Δτ_*y*_ ∈ [3.5 h, 4.5 h] and Δτ_*z*_ ∈ [1.5 h, 2.5 h], the optimal set of phase differences giving the minimum cost function was selected. Finally, the peak expression time of *Per3* was estimated as CT_Per3_ = 24(0.5π-*θ*)/(2π). To evaluate the cosine curve fitting, we computed the coefficient of determination *r*^2^ between the experimental measurements and cosine curves. We also calculated 95% confidence intervals for the estimated value from the inverse of the Hessian matrix of the cost function, based on the assumption that the estimation errors are uncorrelated and normally distributed. The time intervals of hair sampling were not identical among subjects but varied by a maximum of about ±1 h. However, we previously compared the difference in phase-estimation accuracy among various combinations of three data points^28^. Our mathematical analysis indicated high estimation accuracy for various sampling intervals (e.g., 8 h–8 h, 6 h–6 h and 3 h–3 h intervals). Small differences in sampling interval are therefore not expected to affect phase-estimation accuracy.

### Animals

*Per2-luciferase* knock-in mice (*Per2*^*Luc*^ mice) were a kind gift from Dr. Joseph Takahashi^33^. Mice were maintained on a 12-h/12-h light dark cycle (light on at 9:00 a.m.) and allowed *ad libitum* access to food and water. All protocols for animal experiments were approved by the Animal Research Committee of Yamaguchi University. Animal studies were performed in compliance with the Yamaguchi University Animal Care and Use guidelines.

### Explant cultures and bioluminescence measurement

Coronal brain slices including the SCN (300–400 μm thickness) were prepared from adult *Per2*^*Luc*^ mice using a vibratome^34,35^. Paired SCNs were excised from coronal brain slices and placed on a culture membrane (Millicell-CM; Merck, Germany) in a covered and sealed culture dish filled with medium containing 100 μM luciferin. Bioluminescence was measured in realtime with a photomultiplier tube (LM2400; Hamamatsu, Japan). The data sets were detrended by subtracting the 24 h running average from the raw data. To perform single neuron imaging, a luminescence microscope optimized for live cell imaging (LV200; Olympus, Japan) was used.

## Results

**Figure 1:**
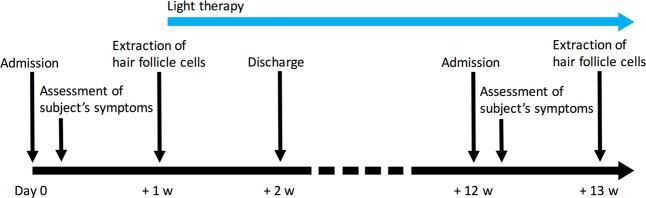
Experimental Design. Japanese PD patients were exposed to a single period of one hour of light at 5,000 lux once a day between 19:00–21:00. This bright light exposure was performed every day for a period of approximately three months in total: one week during hospitalization, more than 10 weeks at home after discharge and one week during re-hospitalization (blue arrow). Before and after the period of bright light exposure, patients completed the UPDRS part III, ESS and PDSS-2, and hair follicles were collected from each patient. w represents week(s).

Although BLT reportedly improves sleep in PD patients receiving DT, as described above, the number of participants and trials examined and research groups to have investigated the efficacy to date has been small. Further studies are therefore required to confirm the clinical usefulness of BLT. In the present study, we investigated the effect of BLT on sleep problems in East Asian (Japanese) PD patients. Given that PD patients receiving DT show early-morning awakening and circadian phase advance of melatonin secretion, we exposed patients to one hour of bright light once a day during the evening hours (Fig. 1). This bright light exposure was performed every day for a total period of approximately three months. PD patients participating in this study were aged 52–80 years, all of whom were receiving DT and had a UPDRS part III score of 9–45 (Table 1). Sleep was evaluated using the Epworth Sleepiness Scale (ESS) and Parkinson Disease Sleep Scale 2 (PDSS-2). Re-examination of these scales after the period of bright light exposure revealed no significant change in the UPDRS part III or ESS scores but showed a significant positive impact on sleep according to the PDSS-2 score (Table 2). Statistical comparison for each item of PDSS-2 between before and after BTL revealed a significant improvement in two items: nocturia and pain in arms or legs. To examine the effect of aging on BLT efficacy, we divided the PD patients into younger and older groups (n = 8, each) and performed the statistical analysis again. Interestingly, although the sample size is likely too small to make solid conclusions, the results suggest that BLT induced a statistically significant improvement in PDSS score only in the younger group. The disappearance of statistical significance for nocturia and pain in arms or legs is probably due to the reduced sample size after grouping. Together, these results show that BLT is a promising therapy for sleep problems in PD patients receiving DT.

**Table 1:** Patients’ clinical details.

Patient	Age (yrs)	Gender	Disease duration (yrs)	UPDRS part III score	ESS score	PDSS-2 score	Medication*
#1	62	M	4	29	8	15	Rop 2 mg; C/L 30/300 mg
#2	65	M	18	21	8	24	C/L 50/500 mg; E 300 mg; Z 50 mg; T 4 mg; A 150 mg
#3	70	F	27	19	6	37	Rop 2 mg; C/L 60/600 mg; E 400 mg; S 2.5 mg; A 100 mg
#4	61	F	8	15	10	21	Rop 4 mg; C/L 20/200 mg; S 2.5 mg
#5	71	F	15	35	12	40	Pra 1.875 mg; C 3 mg; C/L 40/400 mg; A 100 mg; E 400 mg
#6	64	F	15	26	14	14	Pra 0.375 mg; Rot 13.5 mg; C/L 40/400 mg; A 150 mg; E 400 mg; S 5 mg
#7	61	F	13	39	2	20	Rop 8 mg; Rot 18 mg; C/L 70/700 mg; E 700 mg
#8	52	F	4	24	13	14	C/L 30/300 mg
#9	65	F	1.5	29	4	16	C/L 10/100 mg; T 2 mg
#10	54	M	17	9	9	17	C/L 40/400 mg; S 2.5 mg; T 10 mg
#11	66	F	8	26	11	8	Pra 1.5 mg; B/L 87.5/350 mg; E 200 mg; S 2.5 mg
#12	77	M	3.5	43	4	28	C/L 35/350 mg; Z 25 mg; A 50 mg
#13	66	F	11	45	7	25	Rot 22.5 mg; C/L 50/500 mg; E 500 mg; S 5 mg; Z 25 mg
#14	80	M	20	32	8	19	C/L 30/300 mg
#15	67	M	6	28	11	12	Rot 6.75 mg; C/L 20/200 mg
#16	66	F	7	39	0	19	Pra 0.375 mg; Z 25 mg; C/L 40/400 mg; E 300 mg

Abbreviation: UPDRS, Unified Parkinson Disease Rating Scale; ESS, Epworth Sleepiness Scale; PDSS, Parkinson’s Disease Sleep Scale.

*Rop, Ropinirole; C/L, carbidopa/levodopa; E, entacapone; Z, zonisamide; T, trihexyphenidyl; A, amantadin; S, selegiline; Pra, pramipexole; C, cabergoline; Rot, Rotigotine; B/L, benserazide/levodopa.

**Table 2:** Effects of BLT on disease severity and sleep in PD patients.

		Mean (SD)		P Value*All/Younger/Older
		Before BLT	After BLT	
UPDRS part III score		22.31 (8.90)	22.64 (8.33)	0.970/0.599/0.792
ESS score		7.94 (3.97)	7.56 (3.61)	0.663/0.492/0.958
PDSS-2 score		20.56 (8.65)	14.56 (7.87)	0.038/0.007/0.371
Items	#1 Overall sleep	2.19 (1.38)	1.44 (1.26)	0.098/0.045/0.553
	#2 Difficulty falling asleep	1.88 (1.31)	1.13 (1.36)	0.091/0.615/0.554
	#3 Difficulty staying asleep	3.00 (1.59)	2.88 (1.31)	0.662/0.601/0.954
	#4 Restlessness of legs or arms	1.44 (1.36)	0.75 (1.39)	0.068/0.077/0.462
	#5 Urge to move legs or arms	1.13 (1.36)	1.06 (1.48)	0.770/0.502/0.909
	#6 Distressing dreams	0.63 (1.02)	0.38 (0.62)	0.730/0.350/0.573
	#7 Distressing hallucinations	0.63 (1.20)	0.25 (0.45)	0.582/1.00/0.488
	#8 Nocturia	3.25 (1.13)	2.38 (1.31)	0.044/0.253/0.103
	#9 Immobility in bed	1.44 (1.71)	0.94 (1.29)	0.473/0.856/0.508
	#10 Pain in arms or legs	1.19 (1.52)	0.19 (0.40)	0.019/0.052/0.212
	#11 Muscle cramps in arms and legs	0.75 (1.34)	0.38 (1.03)	0.261/0.349/0.587
	#12 Morning dystonia	0.75 (1.24)	0.63 (1.09)	0.913/ 1.00/0.904
	#13 Morning tremor	0.31 (0.60)	0.25 (0.68)	0.488/0.700/0.643
	#14 fatigue and sleepiness in the morning	1.75 (1.44)	1.31 (1.25)	0.394/0.698/0.546
	#15 Snoring and difficulties of breathing	0.25 (0.58)	0.44 (1.09)	0.934/0.587/0.706

*All: All subjects (n = 16), Younger: Subjects between the ages of 52 and 65 (n = 8), Older: Subjects between the ages of 66 and 80 (n = 8).

**Figure 2:**
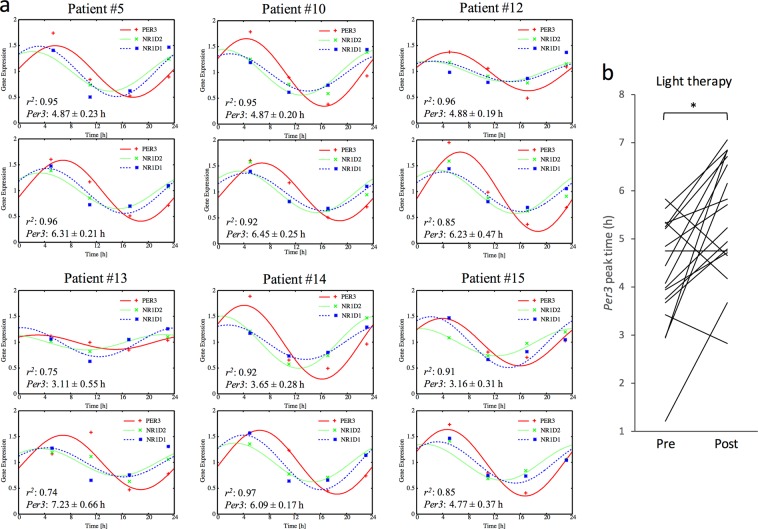
Effect of BLT on peripheral clock gene expression in PD patients receiving DT. (**a**) Representative clock gene expression rhythms of PD patients receiving DT before and after BLT (upper and lower panels, respectively). Hair follicles were collected at approximately 6‐hr intervals around the clock, from which total RNA was extracted and subjected to real‐time PCR for quantification of the expression levels of three clock genes (*Per3*, *Nr1d1* and *Nr1d2*). Expression levels were normalized based on the expression level of *18S ribosomal RNA*. A mathematical estimation of peripheral circadian phase was performed based on the expression levels of these three clock genes. Colored curves and dots represent estimation results and experimental measurements, respectively. Estimated peak times in *Per3* expression rhythms are shown with the coefficient of determination *r*^2^ and 95% confidence interval. (**b**) Statistical comparison of *Per3* peak times in PD patients receiving DT before or after BLT using a paired Student’s t-test (n = 17, *P < 0.01).

To our knowledge, no study has reported experimental data on the physiological mechanism by which BLT improves sleep problems in PD patients. Sleep is regulated by both homeostatic and circadian function^36–38^. Based on the hypothesis that BLT improves sleep via the circadian pathway, we evaluated the effect of BLT on circadian function in PD patients. Although it would be optimal to functionally evaluate the SCN, the central clock located in the hypothalamus, in PD patients to examine this aim, direct detection and evaluation of SCN function is technically unrealistic at present. We therefore investigated the effect of BLT on circadian function by measuring and evaluating peripheral clock gene expression using hair follicles (Fig. 2). The schedule for hair follicle collection before and after BLT is shown in Fig. 1. Representative clock gene expression rhythms of six PD patients receiving DT are shown in Fig. 2a. We semi-quantified the expression levels of three clock genes (*Per3*, *Nr1d1* and *Nr1d2*). According to previous reports that showed that sampling at 6‐hr intervals is sufficient to reliably estimate the circadian phase^28,31^, we performed a mathematical estimation of the peripheral circadian phases based on the expression levels of the three clock genes. As a control experiment, peripheral circadian phase estimation was also performed in non-PD elderly subjects aged 59–82 years who had stayed in a rehabilitation hospital over a period of at least two weeks (Fig. S1). Although light-dark and eating schedules are known to be potent cues for circadian entrainment, these parameters were almost identical between the non-PD and PD subjects (see Materials and Methods). These data indicate that PD patients show a similar distribution in peripheral circadian phases to non-PD subjects. Comparison of *Per3* peak times between before and after BLT (Fig. 2a, upper and lower panels, respectively) revealed a delay in the peripheral circadian phase. The coefficient of determination *r*^2^ and 95% confidence interval for estimated peak times of *Per3* are presented in the figure as indices to indicate successful cosine curve fitting. As expected from a standard human circadian phase response curve to light input^39,40^, these results indicate that clock gene expression rhythms showed a circadian phase delay in 78% of the PD patients who received BLT during the late evening hours (Fig. 2b). Together, these results indicate that BLT indeed acts on the circadian clock of PD patients receiving DT and modulates its functional properties.

**Figure 3:**
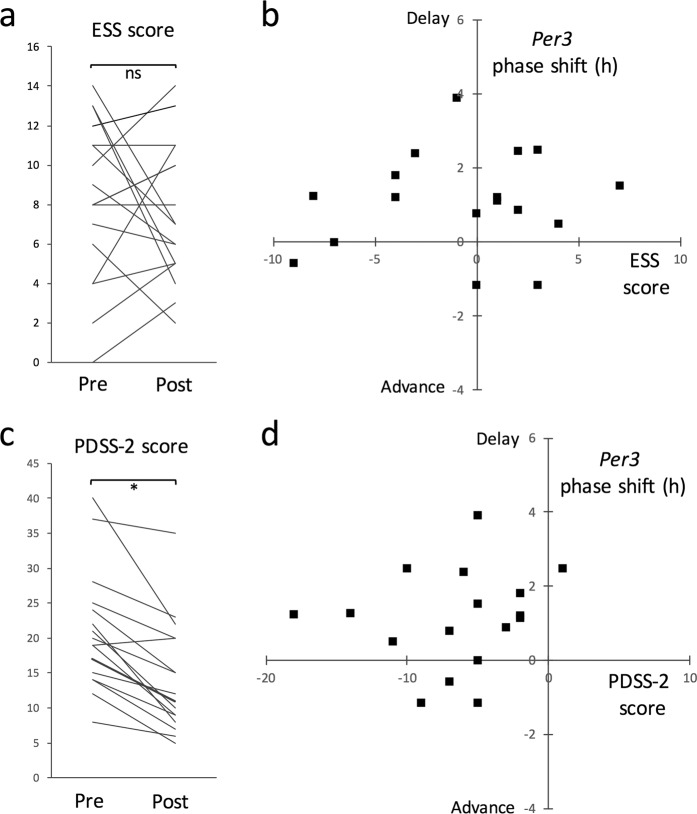
Association between circadian modulation and sleep improvement in PD patients who received BLT. Correlation between BLT-induced changes, namely the change in circadian phase in clock gene expression versus the change in severity in sleep problems was examined. (**a**) Statistical comparison of ESS scores in PD patients receiving DT before or after BLT using a paired Student’s t-test (n = 17; ns, no statistical difference). (**b**) A two-dimensional plot of the change in ESS score versus the change in circadian phase in *Per3* expression. (**c**) Statistical comparison of PDSS-2 scores in PD patients receiving DT before or after BLT using a paired Student’s t-test (n = 17, *P < 0.01). (**d**) A two-dimensional plot of the change in PDSS-2 score versus the change in circadian phase in *Per3* expression.

Next, to evaluate the association between circadian modulation and sleep improvement in PD patients who received BLT, we examined the correlation between BLT-induced changes, namely, the change in circadian phase in clock gene expression versus the change in severity in sleep problems (Fig. 3). No significant improvement in sleep was detected using ESS in PD patients who received BLT (Fig. 3a), and a two-dimensional plot of the change in ESS score versus the change in circadian phase in clock gene expression showed no clear correlation between these parameters (Fig. 3b). In contrast, a significant improvement in sleep was detected using PDSS-2, with the exception of one patient (Fig. 3c). Further, a two-dimensional plot of the change in PDSS-2 score versus the change in circadian phase in clock gene expression showed a clear correlation between these parameters (Fig. 3d): 75% of PD patients with sleep improvement showed a circadian phase delay in clock gene expression. Although the causal link remains unclear, we speculate that functional modulation of the circadian clock is a potential mechanism underlying the BLT-mediated sleep improvement in PD patients receiving DT.

**Figure 4:**
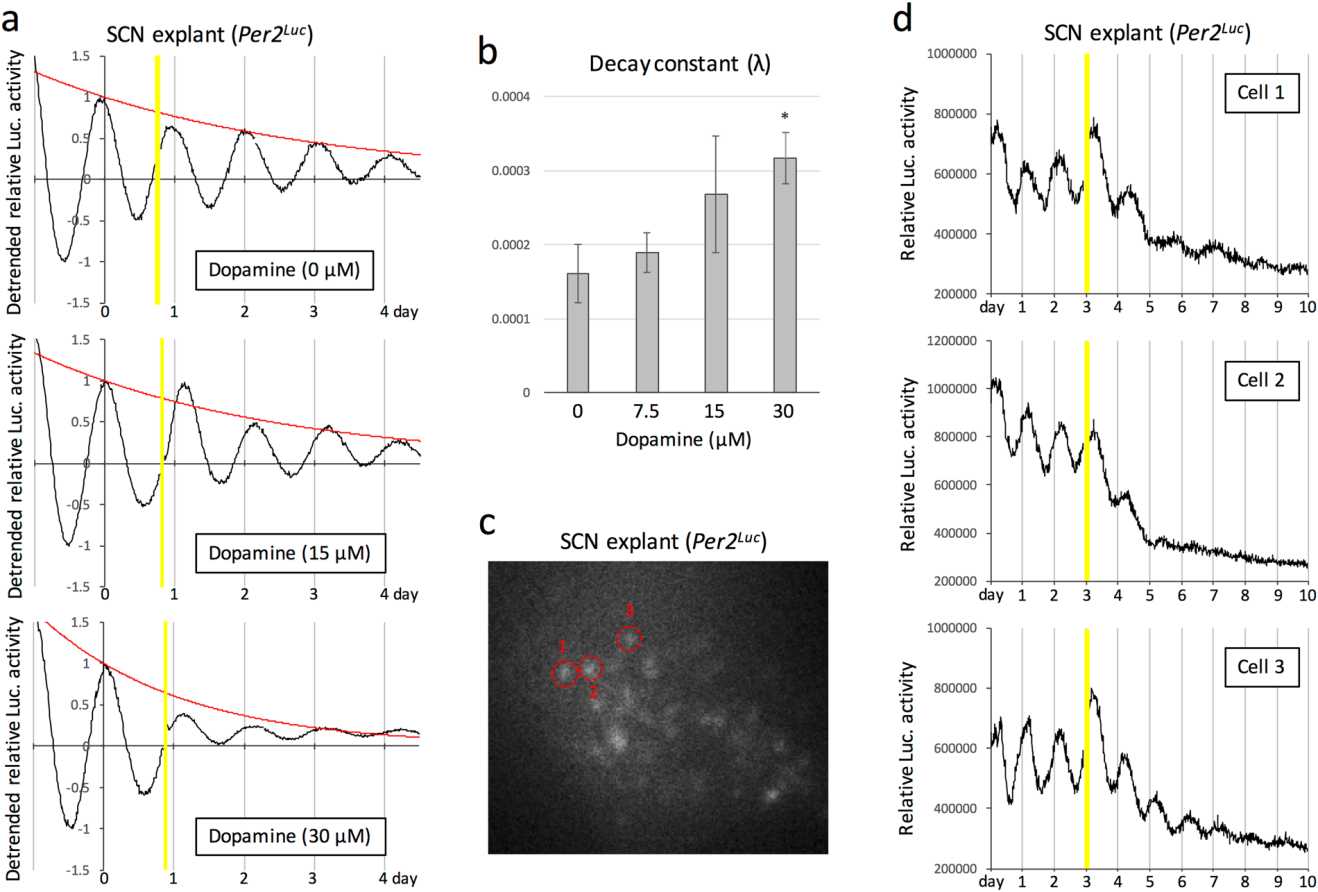
Effect of chronic dopamine exposure to the SCN on circadian gene expression. (**a**) Representative bioluminescence rhythms of *ex vivo* cultured SCN of *Per2*^*Luc*^ mice in the presence of indicated concentrations of dopamine. Bioluminescence was measured in real time with a photomultiplier tube, in the presence of 100 μM luciferin. The data sets were detrended by subtracting the 24-h running average from the raw data. Yellow shadows indicate the time spent for dopamine administration. To calculate the decay constant (λ), peak values were fitted to the model equation y(t) = e^−λt^ by a least-squares method (red curve). (**b)** Statistical evaluation of decay constants (λ) in circadian clock gene expression using an unpaired Student’s t-test (versus 0 μM control, *P < 0.05). (**c**) A representative image of *ex vivo* cultured SCN obtained using a luminescence microscope optimized for single-cell imaging. Red circles surrounding individual neurons indicate ROI (region of interest), which was used for quantification of signal intensity in (**d**). (**d**) Results from real-time and single-cell imaging of *ex vivo* cultured SCN in the presence of 30 μM dopamine. The intensity of bioluminescence emitted from each neuron was integrated for 15 min at intervals of 15 min. Yellow shadows indicate the time spent for dopamine administration.

The hypothalamic SCN of PD patients receiving DT is chronically exposed to dopaminergic stimulation. To experimentally evaluate the possible effect of chronic dopaminergic stimulation on the function of the SCN, we examined the effect of chronic dopamine exposure to *ex vivo* cultured mouse SCN on circadian clock gene expression (Fig. 4). Autonomous circadian rhythms of the expression of the circadian protein Period2 (Per2) in *ex vivo* cultured SCN were detectable in real time using knock-in mice expressing Per2 fused to firefly luciferase (*Per2*^*Luc*^ mice). Chronic exposure of the cultured SCN to dopamine induced a slight and strong attenuation of bioluminescence rhythms at concentrations of 15 and 30 μM, respectively (Fig. 4a). A decay constant was calculated to evaluate the statistical significance of the attenuation of bioluminescence rhythms, and a significant attenuation was confirmed at a concentration of 30 μM (Fig. 4b). To clarify whether this attenuation was attributable to desynchronization of circadian rhythms among SCN neurons or dampening of circadian oscillations in individual neurons, we examined the effect of chronic exposure to dopamine on the circadian rhythms of bioluminescence using highly sensitive imaging at the single neuron level (Fig. 4c). The data clearly demonstrated that chronic exposure of the cultured SCN to 30 μM dopamine caused a rapid attenuation of bioluminescence rhythms in individual neurons (Fig. 4d). Together, these results suggest that functional defects in the SCN caused by chronic exposure to dopamine may in part underlie the sleep problems observed in PD patients receiving DT.

## Discussion

At present, there is no evidence-based international consensus or guideline for medical treatment of sleep problems in PD patients. These sleep problems, coupled with the adverse effects of medications prescribed for PD, limit the usefulness of available treatment strategies. Non-pharmacological treatments for sleep problems are highly desirable because additional pharmacological interventions may cause unwanted side effects via unpredictable interactions with dopaminergic drugs. In the present study, we demonstrated that BLT, a non-pharmacological and chronobiological treatment, was highly effective for improving sleep in Japanese PD patients receiving DT. Although objective scales such as polysomnography are more informative for sleep evaluation, PD patients often feel uncomfortable during sleep. We therefore used subjective scales such as ESS and PDSS-2 in this study, which have been proven to be excellent for assessing sleep in PD patients^41,42^. BLT is relatively easy to prescribe and incorporate into clinical practice, with patients simply required to expose themselves to bright light for one hour once a day. Patients can therefore simply and safely continue the treatment at home after discharge from hospital. Indeed, in the present study, patients performed BLT by themselves at home during the major part of the experimental period. Therefore, BLT is a promising treatment for sleep problems in PD patients receiving DT.

To establish and generalize BLT-based therapies in clinical settings, it is indispensable to elucidate and understand the mechanism by which BLT improves sleep in PD patients receiving DT. However, no reports have suggested potential mechanisms based on experimental evidence. According to the two-process model, sleep is regulated by both homeostatic function and the circadian clock: while homeostatic sleep pressure (process S) increases with time awake and declines during sleep, alertness levels are dependent on the internal circadian clock (process C)^36–38^. The interaction between these two processes generates the diurnal rhythms of the sleep-wake cycle. Because the circadian clock is functionally modulated by retinal light input, there has been speculation, although still lacking sufficient experimental evidence, that the circadian clock is part of the mechanism by which BLT improves sleep in PD patients^16^. In the present study, we successfully provided supportive evidence for this hypothesis. Briefly, experimental evaluation of peripheral clock gene expression rhythms revealed that most of the PD patients receiving DT who experienced improved sleep following BLT indeed showed a circadian phase shift, indicating the existence of a correlation between circadian modulation and sleep improvement. While these results do not demonstrate a causal link between circadian modulation and sleep improvement, they provide experimental evidence to support the hypothesis that BLT improves sleep via circadian function.

Conversely, if this hypothesis holds true, sleep problems in PD patients receiving DT should arise at least in part as a result of circadian dysfunction. By conducting animal experiments in the present study, we successfully demonstrated the possibility that PD patients receiving DT may indeed exhibit circadian dysfunction. Because almost all PD patients chronically receive dopaminergic drugs, the SCN of the hypothalamus is exposed to chronic dopaminergic stimulation. Given that dopamine is released into the synaptic cleft at several tens to hundreds of micromolar concentrations, we chronically administered 30 μM (or less) dopamine to *ex vivo* cultured SCN to experimentally reproduce this *in vivo* situation and examine the effect on SCN function. We found that chronic dopaminergic stimulation induced a rapid attenuation of autonomous oscillations of clock gene expression in the SCN at the single neuron level. Importantly, our findings are consistent with those of a recent study. Following expression of the D1 dopamine receptor (Drd1) in the SCN, Grippo *et al*. found that selective activation of dopaminergic input to the SCN resulted in acceleration of the circadian phase shift, and mice lacking the functional *Drd1* gene showed defects in the light-induced circadian phase shift^20^. Light-induced dopamine release therefore plays a physiological role in modulating SCN function, while DT-induced artificial and chronic dopaminergic stimulation may cause functional defects in the SCN. Indeed, not only decreased amplitude of the circadian rhythms of melatonin, an endocrine marker of circadian rhythms in the SCN, but also altered clock gene expression in peripheral blood mononuclear cells suggests functional defects in the SCN in PD patients receiving DT^8,9^. Given that regular and timed light input resets and potentiates suprachiasmatic circadian rhythms, these findings suggest that BLT may restore SCN function disturbed by DT, which in turn improves sleep. Although we performed BLT during the late evening hours in the present study, BLT scheduled at other times of day may also be effective for restoring SCN function: a report demonstrated improved sleep in PD patients who received one hour of BLT twice a day (in the morning at 9–11 AM and in the afternoon at 5–7 PM)^16^.

In conclusion, we demonstrated that BLT is a promising medical treatment for improving sleep in PD patients receiving DT and that this BLT-induced sleep improvement may arise due to restoration of circadian function. Further studies such as those that aim to determine the optimal time of day and minimum duration of bright light exposure, and the optimal illumination intensity and wavelength of irradiation light are required to increase the clinical usefulness of BLT. In addition, development of inexpensive wearable devices for bright light exposure will improve the generalizability of BLT. For example, while we used a table-top illuminator in the present study, low-cost and glasses-type wearable devices will increase compliance to the therapy in a homecare setting due to reduced limitations to patients’ behavior.

## Supplementary information

Supplementary Information.
