# Supplementary material for: Bright light improves sleep in patients with Parkinson’s disease: possible role of circadian restoration
Source: Sci Rep. 2020 May 14;10:7982. doi: 10.1038/s41598-020-64645-6 (PMC7224174; doi:10.1038/s41598-020-64645-6)
Supplement: Supplementary file 1 — Supplementary Information. [file 41598_2020_64645_MOESM1_ESM.pdf]

Supplementary information

Bright light improves sleep in patients with Parkinson's disease: possible role of circadian restoration

Takuyuki Endo [a], Ritsuko Matsumura [b], Isao Tokuda [c], Tomoko Yoshikawa [d], Yasufumi Shigeyoshi [d], Koichi Node [e], Saburo Sakoda [a, f] and Makoto Akashi [b*]

a) Department of Neurology, Osaka Toneyama Medical Center, 5-1-1 Toneyama, Toyonaka, Osaka 560-8552, Japan
b) The Research Institute for Time Studies, Yamaguchi University, 1677-1 Yoshida, Yamaguchi, Yamaguchi 753-8511, Japan
c) Department of Mechanical Engineering, Ritsumeikan University, 1-1-1 Nojihigashi, Kusatsu, Shiga 525-8577, Japan
d) Department of Anatomy and Neurobiology, Kindai University, 377-2 Ohno-Higashi, Osaka-Sayama, Osaka 589-8511, Japan
e) Department of Cardiovascular Medicine, Saga University, 5-1-1 Nabeshima, Saga, Saga 849-8501, Japan
f) Organic Clinic, 3-1-57 honmachi, Toyonaka, Osaka 560-0021, Japan

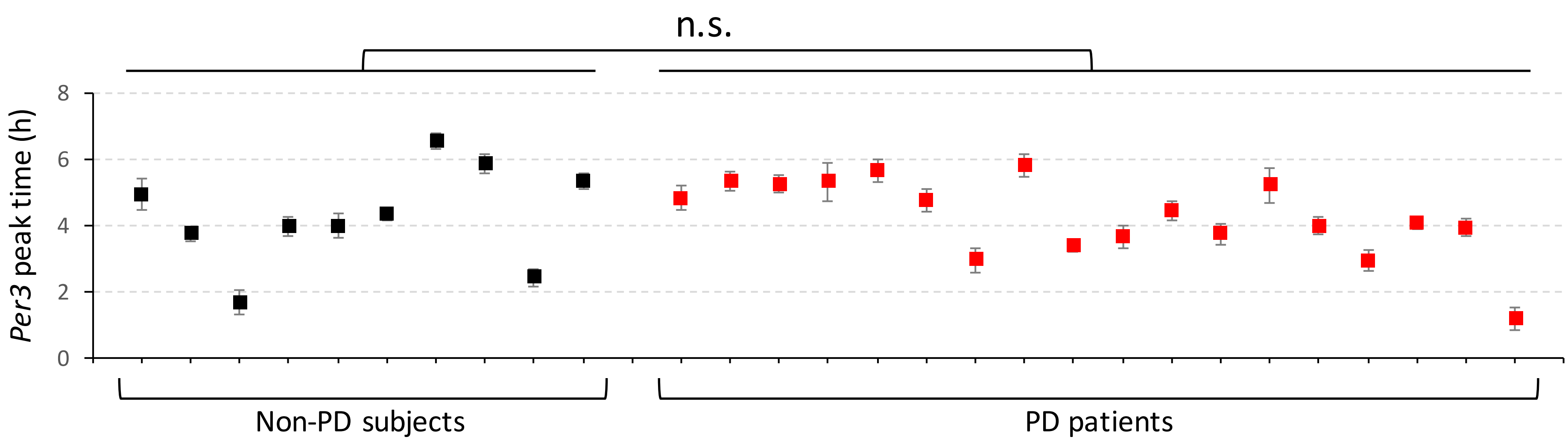

Figure S1 Comparison of peripheral circadian phase between non-PD and PD subjects
The estimated peak time of *Per3* expression in hair follicles was compared between 10 non-PD elderly subjects (black squares; age, 71.4 ± 7.4; sex, male 40%) and 18 PD patients (red squares; age, 66.2 ± 7.5; sex, male 33.3%). Error bars indicate 95% confidence intervals. n.s. represents no statistical significance between the two groups.
